# Polycyclic Aromatic Hydrocarbons (PAHs) Exposure Triggers Inflammation and Endothelial Dysfunction in BALB/c Mice: A Pilot Study

**DOI:** 10.3390/toxics10090497

**Published:** 2022-08-27

**Authors:** Gabriel A. Rojas, Nicolás Saavedra, Kathleen Saavedra, Montserrat Hevia, Cristian Morales, Fernando Lanas, Luis A. Salazar

**Affiliations:** 1 Center of Molecular Biology & Pharmacogenetics, Department of Basic Sciences, Scientific and Technological Bioresource Nucleus (BIOREN), Universidad de La Frontera, Temuco 4811230, Chile; 2 Faculty of Health, School of Kinesiology, Universidad Santo Tomás, Valdivia 5090000, Chile; 3 Department of Internal Medicine, Faculty of Medicine, Universidad de La Frontera, Temuco 4811230, Chile

**Keywords:** air pollution, endothelium, inflammation, cardiovascular disease

## Abstract

The particulate matter present in air pollution is a complex mixture of solid and liquid particles that vary in size, origin, and composition, among which are polycyclic aromatic hydrocarbons (PAHs). Although exposure to PAHs has become an important risk factor for cardiovascular disease, the mechanisms by which these compounds contribute to increased cardiovascular risk have not been fully explored. The aim of the present study was to evaluate the effects of PAH exposure on systemic pro-inflammatory cytokines and markers of endothelial dysfunction. An intervention was designed using a murine model composed of twenty BALB/c male mice separated into controls and three groups exposed to a mixture of phenanthrene, fluoranthene, and pyrene using three different concentrations. The serum levels of the inflammatory cytokines and gene expression of adhesion molecules located on endothelial cells along with inflammatory markers related to PAH exposure in aortic tissue were determined. Furthermore, the expression of the ICAM-1 and VCAM-1 proteins was evaluated. The data showed significant differences in IL-6 and IFN-γ in the serum. In the gene expression, significant differences for ICAM-1, VCAM-1, and E-Selectin were observed. The results suggest that phenanthrene, fluoranthene, and pyrene, present in air pollution, stimulate the increase in serum inflammatory cytokines and the expression of markers of endothelial dysfunction in the murine model studied, both relevant characteristics associated with the onset of disease atherosclerosis and cardiovascular disease.

## 1. Introduction

Air pollution is a serious global public health problem. According to the Ambient Air Quality Guide of the World Health Organization (WHO), 95% of the world population lives in areas exceeding the recommended values [1]. The most recent estimates revealed that 4.2 million deaths (7.6% of total global mortality and 700,000 more deaths in 2015 compared with 1990) are attributable to ambient particulate matter_2.5_ (PM_2.5_) [2]. PM is a widespread complex mixture of solid and liquid particles suspended in air that vary in size, shape, origin, and composition. PM is an air pollutant known as a human carcinogen (group I, IARC, 2013). The composition of PM can substantially vary between geographical regions, sources of emissions, and even weather or seasons [3]. Its chemical composition comprises inorganic ions (e.g., sulfates, nitrates, ammonium, and soluble metals), insoluble metals, elemental carbon, and organic compounds including PAHs, polychlorinated biphenyls, biological components (allergens), microbial agents, and water. The carbonaceous part of air pollution is regarded as more involved in adverse health effects, and some PAHs are considered as particularly important [4]. Thus, PM and PAH are among the most health-relevant air pollutants [5,6].

The three main ways in which air pollution causes damage to the cardiovascular system have been proposed: (a) secretion of pro-inflammatory mediators or oxidative stress in the circulatory system; (b) imbalance of the autonomic nervous system; and (c) direct penetration of particles or components in the circulatory system, which affect numerous tissues within the cardiovascular system [7]. Reports indicate that the smallest particles increase the risk of cardiovascular events, with PM_2.5_ being specifically associated with an increased risk of myocardial infarction, stroke, arrhythmia, and exacerbation of heart failure symptoms in susceptible patients [8].

PM accumulation, especially redox active components (e.g., metals and PAHs), can cause oxidative stress and inflammation in lung tissue [9]. The inflammatory response to exposure to PM is characterized by the increased expression of pro-inflammatory cytokines such as tumor necrosis factor alpha (TNF-α) and interleukin-6 (IL-6), which are secreted by cells of the innate immune system [10]. In addition, the adaptive immune system releases interleukin-1β (IL-1β), interleukin-4 (IL-4), and IL-6 [11]. These cytokines are released into the circulatory system, increasing the liver production of C-reactive protein (CPR) and fibrinogen, IL-6, IL-1β, interferon-gamma (IFN-γ), interleukin-8 (IL-8), and TNF-α [8]. This increase in cytokines associated with exposure to PM has been described in various studies [12,13,14,15].

Pathological stimuli in the endothelium trigger a phenotype-modifying adaptive response, a process known as endothelial activation, characterized by increased expression of adhesion molecules Selectin-P (P-Selectin), Selectin-E (E-Selectin), intercellular adhesion molecule 1 (ICAM-1), and vascular cell adhesion molecule 1 (VCAM-1) [16,17]. This process compromises the barrier function of the endothelium, which promotes leukocyte diapedesis, increases vascular tone by decreasing nitric oxide production, and reduces resistance to thrombosis [18,19].

Among the toxic components found in PM_2.5_ are PAHs, whose main emission sources are the domestic burning of coal and wood, power plants that burn fossil fuels and biomass, industrial processes, and vehicular traffic [20]. Concentrations of these compounds vary depending on multiple factors such as the season of the year, geographic location, and demographics, among others. Thus, for Temuco, Chile, it was determined that the dominant individual PAHs were phenanthrene (35–45%), fluoranthene (11–15%), and pyrene (9–12%), with the phenanthrene domain reflecting a typical characteristic of emissions from biomass combustion, especially burning wood for heating or cooking [21].

Most of these studies focused their interest on the PM relationship with cardiovascular disease development; however, the mechanisms by which PAH presents in PM contribute to the increased cardiovascular risk have not been explored in depth. Therefore, the objective was to evaluate the effects of PAH exposure on markers of inflammation and endothelial dysfunction in a murine model of BALB/c mice.

## 2. Materials and Methods

### 2.1. Animals

Twenty male BALB/c mice were randomly assigned to four equal groups of five animals each including a control group (C = no exposure) and three groups exposed to 10 µg, 30 µg, and 50 µg of a PAH mixture, composed of 55% phenanthrene (Sigma-Aldrich, St. Louis, MO, USA), 25% fluoranthene (Sigma-Aldrich, St. Louis, MO, USA), and 20% pyrene (Sigma-Aldrich, St. Louis, MO, USA), proportionally to the most representative distribution of the PAHs previously described [21]. Dimethylsulfoxide (DMSO, Sigma-Aldrich, St. Louis, MO, USA) was used as a solvent. The animals were kept in the Bioterio of the University of La Frontera, receiving a standard diet and ad libitum water.

### 2.2. Experimental Protocol

The animals received a 2-week acclimatization treatment according to the instillation protocol described above [22]. The intervention groups underwent nasal instillation of a volume of 10 µL using a micropipette. The intranasal instillation induced an apnea reflex followed by a deep inspiration. In addition, the control group was instilled with the vehicle solution (DMSO) using the same volume. The intervention protocol consisted of instillation for 5 days a week for 5 weeks. We recorded the weight of the animals once a week. To assess the animals’ level of stress and spontaneous activity, the cylinder test was applied every two weeks, where the upright exploration attempts in a transparent cylinder were quantified [23]. Thus, low attempts or immobility indicated the level of activity of the animal. To assess the general condition of the animals, the Morton and Griffiths ‘Animal Supervision Protocol’ was applied once a week [24]. The Scientific Ethics Committee of the Universidad de La Frontera (No 105_18) approved the experimental protocol.

### 2.3. Sampling Extraction

Euthanasia was performed with a mixture of ketamine/xylazine using a lethal intraperitoneal dose of 200 mg/kg of ketamine-16 mg/kg of xylazine. Whole blood sampling was performed by cardiac puncture and centrifuged at 2000 rpm for 15 min. Once the serum was separated from the blood clot, the samples were stored at −80 °C for later analysis. The thoracic aorta was removed and stored at −80 °C in a sterile tube with 1 mL of RNAlaterTM stabilizer solution (Ambion Inc., Austin, TX, USA).

### 2.4. Cytokine Analysis

We analyzed the serum levels of IL-6, IL-10, IL-17A, INF-γ, and TNF-α with the 6-Plex Kit of the Bio-Plex Pro TM Mouse Cytokine Th17 Panel A (BioRad, Hercules, CA, USA) following the manufacturer’s instructions, with the MAGPIX^®^ system (Luminex, Austin, TX, USA). Twenty samples were tested in duplicate in a 96-well plate including an 8-point standard curve in duplicate and two wells as the negative control. Data collection was performed with xPONENT 4.2^®^ software (Luminex, Austin, TX, USA). We adjusted the cytokine values according to the weight of each animal.

### 2.5. Gene Expression by RT-qPCR

Gene expression was analyzed by quantitative real-time polymerase chain reaction (RT-qPCR). Specific primers were used for ICAM-1, VCAM-1, E-Selectin, P-Selectin, platelet endothelial cell adhesion molecule (Pecam-1), endothelial nitric oxide synthase (eNOS), aryl hydrocarbon receptor (Ahr), Kelch-type ECH-associated protein 1 (Keap 1), transcription factor p65 (RelA), inhibitor of nuclear factor kappa-B kinase subunit beta (IKK-β), IL-6, and TNF-α, together with the reference genes for ribosomal protein L32 (RPL32) and beta2-microglobulin (B2M) (Table 1). Frozen aortic tissue samples were lysed using 2 mL prefilled tubes with ceramic beads (MP biomedical, Solon, OH, USA) in a benchtop BeadBugTM homogenizer (Benchmark Scientific, Sayreville, NJ, USA) for 60 s at 3500 rpm, adding 1 mL of TRIzol^®^ reagent (Invitrogen, Waltham, MA, USA). Once the tissue was completely homogenized, the TRIzol^®^ reagent protocol recommended by the manufacturer was followed to extract the total RNA, and subsequent evaluation by spectrophotometry (NanoQuant Infinite^®^ 200 PRO, Tecan^®^, Männedorf, Switzerland) and fluorometry (Quantus™ Fluorometer, Promega, Madison, WI, USA) to determine the purity (260/280 nm ratio) and the amount of RNA extracted, respectively. A ratio between 1.8 and 2.0 was considered as acceptable. The total RNA samples were diluted to ensure a final concentration of 30 ng/µL. The synthesis of cDNA was carried out through reverse transcription using the High-Capacity cDNA Reverse Transcription Kit (Applied Biosystems, Foster City, CA, USA). A qPCR was performed to quantify the expression of each of the selected genes and housekeeping gene using the Fast SYBR^®^ Green Master Mix Kit (Applied Biosystems, Foster City, CA, USA) following the manufacturer’s protocols. For qPCR analysis, LinRegPCR^®^ software was used, which established a linearity window and calculated the PCR efficiencies per sample. With the average PCR efficiency per sample, Ct value, and fluorescence threshold established, the initial concentration per sample expressed in arbitrary fluorescence units was calculated [25]. To analyze the specificity of the primers, the melting curve was evaluated.

### 2.6. Western Blotting

Protein levels of ICAM-1 and VCAM-1 were quantified using α/β-tubulin as a loading control. We performed total protein extraction from aortic tissue using the TRIzol^®^ reagent protocol. Total proteins were quantified using the Pierce BCA Colorimetric Assay Kit (Thermo Scientific ^TM^, Rockford, IL, USA) in a 96-well multiplate, in triplicate. The protein extract was diluted 3:1 in 4× Laemmli sample buffer (Bio-Rad, Hercules, CA, USA) before adding β-mercaptoethanol in a 1:9 ratio (Bio-Rad). The final protein concentration used for the immunodetection of each sample was 40 µg. The samples were denatured at 95 °C for 5 min, and then loaded onto a 4–20% Mini-PROTEAN^®^ TGX ^TM^ electrophoresis gel (Bio-Rad). Afterward, electrophoresis was applied at 100 V for 15 min and then 200 V for 30 min. To differentiate the molecular mass of the bands, the Precision Plus Protein ^TM^ Kaleidoscope standard (Bio-Rad) with a volume of 10 µL was used. The proteins were then transferred to the PVDF immunoblot membrane (Bio-Rad) for 1.5 h at 350 mAmp. Once the transfer was complete, the membrane was stained with Ponceau Red Solution S (Biotium, Fremont, CA, USA) to verify the transfer. Subsequently, the membrane was blocked with 5% NFDM/TBS-Tween for 1 h and then incubated with the primary antibodies at 4 °C overnight according to the manufacturer’s instructions. VCAM-1 (1:1000, 5% BSA, 1X TBS, 0.1% Tween^®^20; Cell Signaling 32653, Danvers, MA, USA), ICAM-1 (1:1000, 5% NFDM, 1X TBS, 0.1% Tween^®^20; Abcam, ab179707), and as the loading control α/β-tubulin (1:1000, 5% BSA, 1X TBS, 0.1% Tween^®^20; Cell Signaling 2148, Danvers, MA, USA). Subsequently, the membrane was washed with TBS-Tween and incubated with the HRP-conjugated secondary antibody (1:3000, 5% NFDM 1X TBS, 0.1% Tween^®^20, goat anti-rabbit IgG; Cell Signaling 7074, Danvers, MA, USA) for 1 h at room temperature. Antigen-antibody binding bands were detected using G: BOX Chemi XRQ (SYNGENE, Frederick, MD, USA) chemiluminescence equipment using the SuperSignal ^TM^ West Femto Maximum Sensitivity Substrate Kit (Thermo Scientific ^TM^, Rockford, IL, USA), following the manufacturer’s recommendations. The densitometric analysis of the bands was performed using the ImageJ 1.51j8 open-source software (https://imagej.nih.gov/ij/index.html (accessed on 22 August 2022), National Institutes of Health, Bethesda, MD, USA)

### 2.7. Statistical Analysis

Data were analyzed using Prism 8.0.2 software (GraphPad, San Diego, CA, USA). The results are ex-pressed as the means ± standard error of the mean. To evaluate the distribution of the values obtained, the Shapiro–Wilk normality test was performed. For the comparison of the groups, Welch’s ANOVA with the Dunnett’s multiple comparisons test or its non-parametric simile Kruskal–Wallis and a multiple comparison analysis were used through Dunn’s test. A two-way ANOVA was used for group comparison analyses with two variables. A *p*-value < 0.05 was established for statistical significance.

## 3. Results

### 3.1. Animals

**Figure 1:**
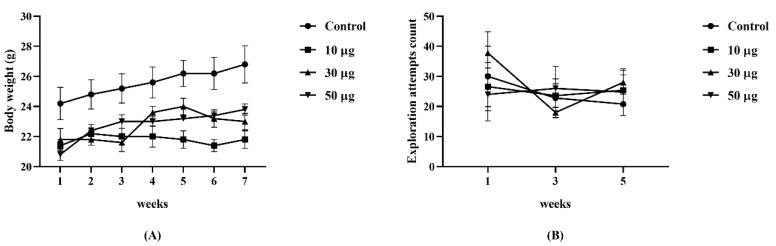
Monitoring of the general condition of the animals. (**A**) Evolution of weight per week by group (*p* = 0.058). (**B**) Comparison of the exploration attempts per group in the cylinder test (*p* = 0.919). Two-way ANOVA. (*n* = 5 per group).

The initial weight per group of animals did not show significant differences (*p* = 0.091). The mean weight was as follows: control = 24.20 ± 1.07 g; group 10 µg = 21.40 ± 0.40 g; group 30 µg = 21.80 ± 0.74 g; group 50 µg = 20.80 ± 0.37 g. The weekly weight of each animal was recorded, which did not show significant differences between the groups (*p* = 0.058). No differences were found in the spontaneous activity of the animals evaluated with the cylinder test (*p* = 0.919; Figure 1).

### 3.2. Serum Cytokines

**Figure 2:**
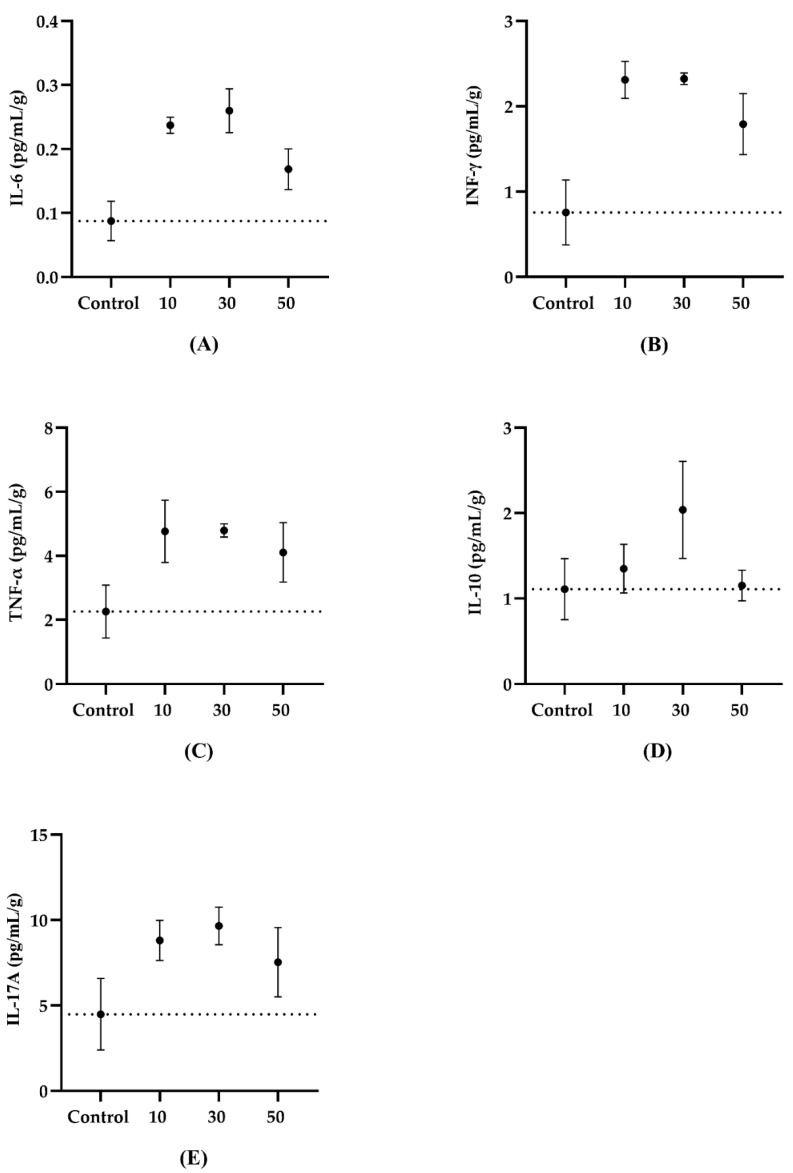
The quantification of weight-adjusted serum inflammatory cytokines in animals exposed to the PAHs and controls. (**A**) IL-6 (*p* = 0.026) [Control v/s 10 µg *p* = 0.025; Control v/s 30 µg *p* = 0.024; Control v/s 50 µg *p* = 0.256]. (**B**) IFN-γ (*p* = 0.039 [Control v/s 10 µg *p* = 0.039; Control v/s 30 µg *p* = 0.050; Control v/s 50 µg *p* = 0.195]). (**C**) TNF-α (*p* = 0.145). (**D**) IL-10 (*p* = 0.576). (**E**) IL-17A (*p* = 0.296). Data are presented as the mean ± SEM. The dashed line represents the mean value of the control group. Welch’s ANOVA with Dunnett’s multiple comparisons test (Control, *n* = 4; 10 µg, *n* = 5; 30 µg, *n* = 4; 50 µg, *n* = 5).

We observed significant differences between the intervention and the control groups for the IL-6 levels (*p* = 0.026) [Control v/s 10 µg, *p* = 0.025; Control v/s 30 µg, *p* = 0.024; Control v/s 50 µg, *p* = 0.256] and IFN-γ (*p* = 0.039) [Control v/s 10 µg, *p* = 0.039; Control v/s 30 µg, *p* = 0.050; Control v/s 50 µg, *p* = 0.195]. In contrast, TNF-α (*p* = 0.145), IL-10 (*p* = 0.576), and IL-17A (*p* = 0.296) did not show differences between the groups (Figure 2).

### 3.3. Gene Expression

**Figure 3:**
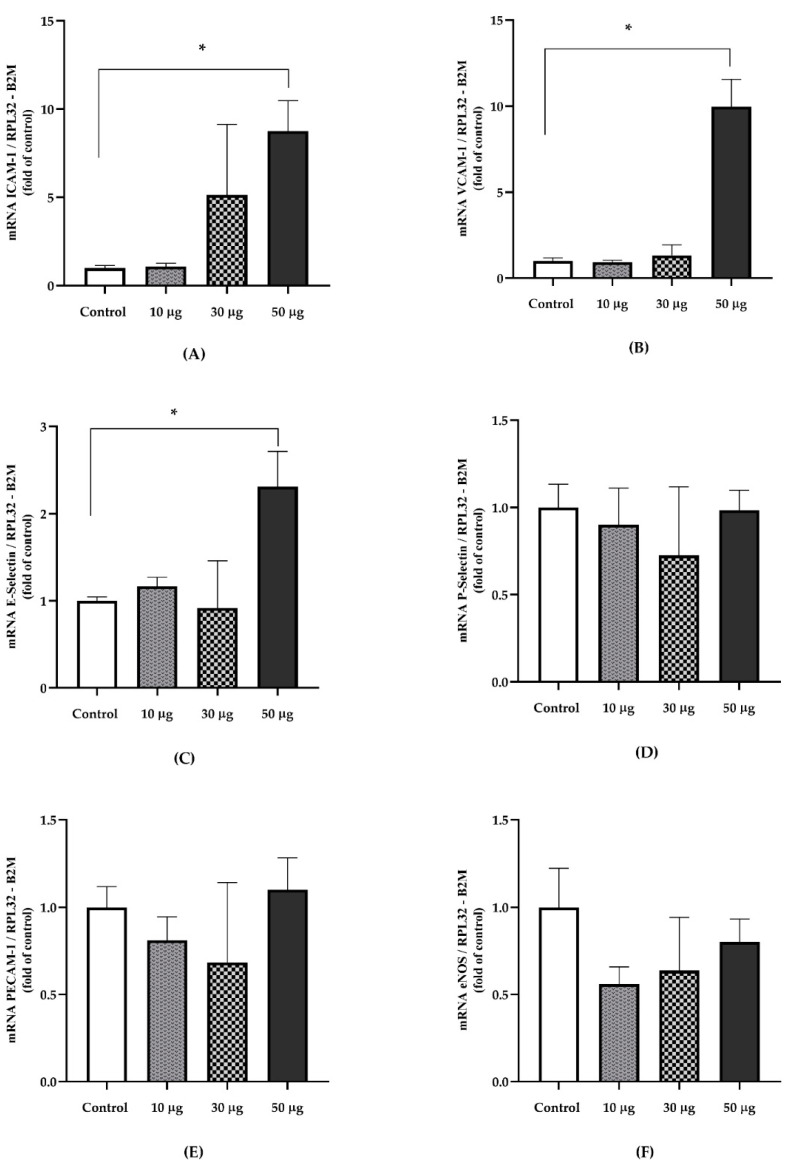
The relative gene expression of endothelial dysfunction markers in the aortic tissue of animals exposed to PAHs and controls. (**A**) ICAM-1 (*p* = 0.047) ^†^ [Control v/s 10 µg, *p* = 0.944]; [Control v/s 30 µg, *p* = 0.655]; [Control v/s 50 µg, *p* = 0.041]. (**B**) VCAM-1 (*p* = 0.023) ^†^ [Control v/s 10 µg, *p* = 0.981]; [Control v/s 30 µg, *p* = 0.910]; [Control v/s 50 µg, *p* = 0.023]. (**C**) E-Selectin (*p* = 0.048) ^‡^ [Control v/s 10 µg, *p* > 0.9999]; [Control v/s 30 µg, *p* > 0.999]; [Control v/s 50 µg, *p* = 0.033]. (**D**) P-Selectin (*p* = 0.986) ^‡^. (**E**) Pecam-1 (*p* = 0.705) ^‡^. (**F**) eNOS (*p* = 0.396) ^†^. Relative quantification was calculated using the reference genes RPL32 and B2M. Data are presented as the means ± SEM. ^†^ Welch’s ANOVA with Dunnett’s multiple comparisons test. ^‡^ Kruskal–Wallis with Dunn’s post hoc analysis (Control, *n* = 4; 10 µg, *n* = 4; 30 µg, *n* = 4; 50 µg, *n* = 4).* *p* < 0.05.

**Figure 4:**
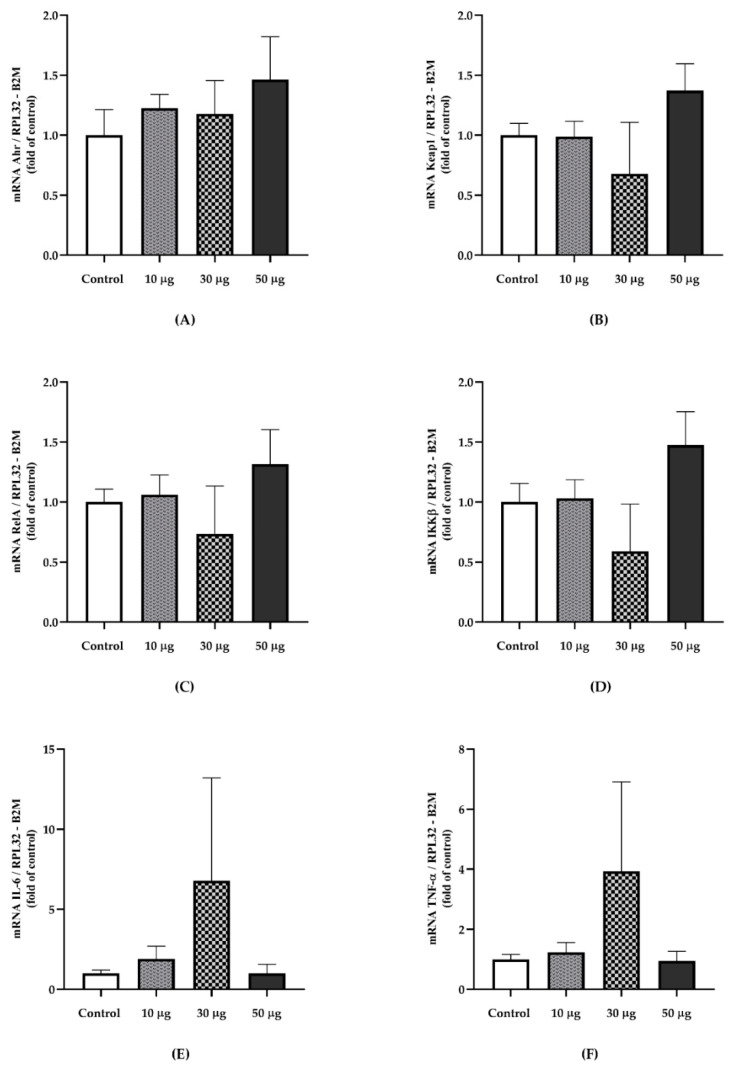
The relative gene expression of exposure-related PAHs and inflammatory markers in aortic tissue from PAH exposed animals and the controls. (**A**) Ahr (*p* = 0.789) ^‡^. (**B**) Keap1 (*p* = 0.507)^‡^. (**C**) RelA (*p* = 0.679) ^‡^. (**D**) IKK-β (*p* = 0.450) ^†^. (**E**) IL-6 (*p* = 0.878) ^‡^. (**F**) TNF-α (*p* = 0.760) ^†^. Gene expression was normalized using the RPL32 and ACTB as reference genes. Data are presented as mean ± SEM. ^†^ Welch’s ANOVA test. ^‡^ Kruskal–Wallis test (Control, *n* = 4; 10 µg, *n* = 4; 30 µg, *n* = 4; 50 µg, *n* = 4).

We observed significant differences for the gene expression of ICAM-1 (*p* = 0.047) [Control v/s 10 µg, *p* = 0,944]; [Control v/s 30 µg, *p* = 0.655]; [Control v/s 50 µg, *p* = 0.041], VCAM-1 (*p* = 0.023) [Control v/s 10 µg, *p* = 0.981]; [Control v/s 30 µg, *p* = 0.910]; [Control v/s 50 µg, *p* = 0.023]; and E-Selectin (*p* = 0.048) [Control v/s 10 µg, *p* > 0.9999]; [Control v/s 30 µg, *p* > 0.999]; [Control v/s 50 µg, *p* = 0.033]. No differences were found for P-Selectin (*p* = 0.986), Pecam-1 (*p* = 0.705), and eNOS (*p* = 0.396) (Figure 3). Regarding markers related to PAH exposure and inflammatory indicators, no differences were identified in Ahr (*p* = 0.789); Keap1 (*p* = 0.507); RelA (*p* = 0.679); IKK-β (*p* = 0.450); IL-6 (*p* = 0.878); TNF-α (*p* = 0.760) (Figure 4).

### 3.4. Protein Expression

**Figure 5:**
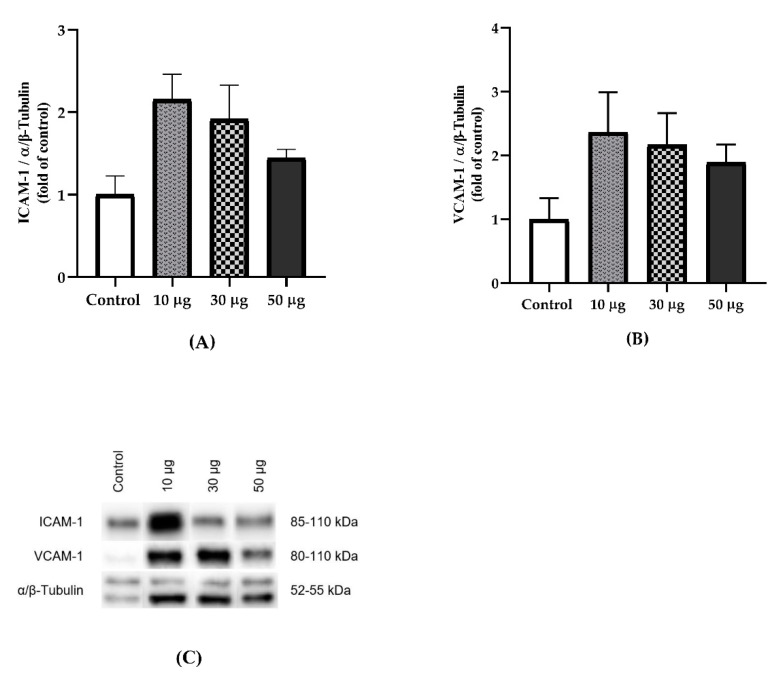
The effect of exposure to PAHs on the protein expression of adhesion molecules in aortic tissue. (**A**) Relative protein expression of ICAM-1 (*p* = 0.117). (**B**) Relative protein expression of VCAM-1 (*p* = 0.210). (**C**) Representative Western blots are shown for ICAM-1, VCAM-1, and α/β-tubulin. Data are presented as the mean ± SEM. Welch’s ANOVA test. (Control, *n* = 4; 10 µg, *n* = 4; 30 µg, *n* = 4; 50 µg, *n* = 4).

The protein expression of ICAM-1 (*p* = 0.117) and VCAM-1 (*p* = 0.210) did not show significant differences in the aortic tissue, although we observed an elevated expression in the intervention groups compared to the control group (Figure 5).

## 4. Discussion

The concentrations and doses of PAHs used for this protocol were established according to previous studies using PM [26] and pollution generated by diesel combustion [27] as we did not find studies using PAH nasal instillation. Some studies have reported that exposure to PAH increases circulating pro-inflammatory cytokines, IL-6, IL-8, and TNF-α being the most studied markers [28,29]. In this study, a significant increase in serum IL-6 and IFN-γ was identified. Another report carried out on workers at a coal plant showed an increased concentration of IL-6 in plasma, demonstrating a dose-dependent relationship with PAH metabolites in urine [28]. IL-6 represents a good indicator of cytokine cascade activation, accurately reflecting the inflammatory state, in addition to its high stability since the half-life of IL-6 is longer than that of other pro-inflammatory cytokines [30]. On the other hand, IFN-γ induces the overexpression of additional pro-inflammatory cytokines such as IL-12, IL-15, TNF-α, IFN-γ-inducible protein-10 (IP-10), inducible nitric oxide synthase (iNOS), among others, inducing the activation of pro-inflammatory transcription factors such as the nuclear factor kappa light chain enhancer of activated B cells (NF-κB) [31]. However, in a study carried out on asthmatic and non-asthmatic children exposed to air pollution from traffic and followed for 6 years, there were no differences in circulating IFN-γ concentrations [32]. However, a study evaluating three different areas of environmental pollution according to their volatile components identified increased IFN-γ and TNF-α concentrations in industrialized and high-traffic areas compared to low-traffic areas [33]. Furthermore, a study evaluating different sources of pollutants derived from wood combustion identified a significant increase in blood TNF-α [34], showing that exposure to particles derived from wood combustion containing PAH can upregulate pro-inflammatory cytokines including IL-6 and TNF-α.

IL-10 has pleiotropic effects on immunoregulation and inflammation, downregulating the expression of Th1 cytokines, class II MHC, and co-stimulatory molecules in macrophages. IL-10 also improves B cell survival, proliferation, and antibody production [35]. This cytokine can block the activity of NF-kB, participating in the regulation of the JAK-STAT signaling pathway. By comparing different pro-inflammatory and anti-inflammatory cytokines associated with different levels of environmental pollution, Dobreva et al. showed that air pollutants, mostly PM_2.5_, modulated cytokine production by altering the TNF-α (pro-inflammatory) and IL-10 (anti-inflammatory) [36].

An increase in the pro-inflammatory cytokines affects the regulation of vascular tone, cell adhesion, inflammation, proliferation, and the phenotype of smooth muscle cells as well as the formation of atheroma plaques [16]. Endothelial activation is characterized by an increase in the expression of adhesion molecules, leukocyte diapedesis, increased vascular tone due to decreased nitric oxide production, and reduced resistance to thrombosis [18]. The process of endothelial activation has been described as the factor initiating atheroma plaques in the vascular tissue. Thus, TNF-α enhances the expression of adhesion molecules in vascular endothelial cells. An in vitro study determined that stimulation of human coronary artery endothelial cells exposed to TNF-α increased ICAM-1 expression [37]. Our data indicate increased expression of the ICAM-1, VCAM-1 and E-Selectin genes, showing significant differences in the group exposed to 50 µg PAHs. Additionally, ICAM-1 and VCAM-1 protein expression was elevated in the PAH-exposed groups, although it did not reach statistical significance, being able to explain this by post-transcriptional regulations that should be studied in more depth.

We also observed a nonsignificant increase in IL-6 and TNF-α. Endothelial activation by TNF-α is carried out by two main mechanisms: activation of NF-κB and MAPK. The activation of NF-kB plays a central role in the regulation of multiple cellular processes such as inflammation, immune response, differentiation, proliferation, apoptosis, and cancer, thus it is considered as a master regulator of inflammatory responses [38]. Furthermore, TNF-α, IL-1β, IL-6, and IFN-γ expression are elevated within the great elastic arteries of old mice and humans [39]. This pro-inflammatory arterial phenotype is associated with increased NF-κB activity. When translocated to the nucleus, NF-κB activates the transcription of genes involved in the production of pro-inflammatory cytokines [40]. As we did not observe an increase in the gene expression of RelA and IKK-β, it becomes necessary to identify the mechanisms by which exposure to PAHs stimulates the increased expression of adhesion molecules in vascular tissue.

Based on the findings of this study, it is necessary to expand the studies that can investigate the mechanisms by which PAHs can generate deleterious effects on the cardiovascular system, being able to speculate that these compounds, due to their nature, manage to enter the cardiovascular system directly via bloodstream, affecting the vascular endothelium manage to enter the cardiovascular system directly via bloodstream, affecting the vascular endothelium. Thus, evidence shows that the passage of small molecules (PM_0.1_) into the blood directly affects the vascular system. Within this group of particles, we can find particles derived from fossil fuels and wood combustion [7,41,42,43]. In this sense, reports have determined that PAHs bind to AhR, leading to the release of the latter from the multiprotein complex and its consequent translocation to the nucleus, where it dimerizes with the nuclear translocator AhR (ARNT), leading to binding to the xenobiotic response element (XRE) in the promoter region of target genes to stimulate transcription [44]. Furthermore, it has been proposed that AhR-mediated pathways are linked to responses to oxidative stress through the dissociation of the nuclear factor erythroid 2-related factor 2 (Nrf2) and the inhibitory protein keap1 [45].

Regarding the behavior of the variables studied, it was originally expected that there would be a dose–response relationship, a situation that was not observed in this investigation. In this regard, it is important to point out that the responses to the different pollutants did not always maintain this behavior, finding little evidence indicating that exposure to increasing doses of PM leads to vasomotor dysfunction and the progression of atherosclerotic plaque [46]. Furthermore, it has been suggested that systemic or pulmonary inflammation is not a prerequisite for dysfunction in the vasomotor response and accelerated the progression of atherosclerosis in animals exposed to PM [46]. Therefore, in the investigation, we wanted to review both the markers of systemic inflammation and the specific effect on tissue, particularly aortic tissue, evaluating markers of endothelial dysfunction that are recognized as potent factors for vasomotor dysfunction and atherosclerotic plaque formation. However, it has been documented that different types of PAH generate effects through different pathways in the tissues. An example of this is benzo[a]pyrene (B[a]P), which is considered to have a low affinity for AhR, but with potent effects on Ca^2+^ induction, a mechanism related to endothelial dysfunction. In contrast, pyrene, which seems to have an even greater effect on Ca^2+^ induction, but a non-nuclear Ahr stimulation pathway [47], showed differences in the effects of various PAHs in their pathophysiological mechanism of action. This is relevant to exemplify that the effect observed in tissue may be influenced by several metabolic pathways, which requires further investigation.

The PAH doses used in this research are within the ranges published in various studies and are adequately summarized in the review by Møller et al. [46]. However, it is important to point out that there is a great variation in the representation of PAHs in the particulate matter in the air, which depends on the concentrations of its different types, emission sources, time of year, place of measurement, and PM level, among others. These factors make direct extrapolation somewhat complex to perform. Thus, for example, residents living in rural areas generally inhale higher concentrations of PM-bound PAHs (4.2–655 ng/m^3^) [48] than residents living in urban areas (0.4–11, 9 ng/m^3^) [49]. In this study, a mixture of phenanthrene, fluoranthene, and pyrene was obtained, which are characteristic of the winter season associated with the combustion of wood for heating and cooking.

Although we have reported interesting findings regarding the role of exposure to PAHs in pro-inflammatory states and cardiovascular health, this study had limitations of a small number of animals per group, considering that no previous data were found regarding the model used for PAH management. In addition, the intervention contemplated 5 weeks of exposure, which could be a bit limited considering the long-term deleterious effects that people exposed to high levels of air pollution have been shown to manifest.

## 5. Conclusions

Our results suggest that PAHs of phenanthrene, fluoranthene, and pyrene, present in PM, partially stimulate the production of serum inflammatory cytokines, which have been associated with the development of various diseases related to high exposure to air pollution, in addition to being a relevant factor for the development of endothelial dysfunction and atherosclerotic disease. Furthermore, an increase in the expression of adhesion molecules related to endothelial dysfunction was found, an initial mechanism in the atherogenesis process that contributes to the formation, progression, and complications of the atherosclerotic plaque, alterations that are usually subclinical and poorly diagnosed. In this studied murine model, we showed that both mechanisms associated with the development of cardiovascular disease were manifested and may represent a model that allows for investigating the cellular and molecular mechanisms associated with exposure to PAHs present in PM.

## Figures and Tables

**Table 1:** The primer sequences used for the PCR analysis.

Gene	Sequence Forward	Sequence Reverse
ICAM-1	TTCTCATGCCGCACAGAACT	TCCTGGCCTCGGAGACATTA
VCAM-1	CTGGGAAGCTGGAACGAAGT	GCCAAACACTTGACCGTGAC
E-Selectin	AGCCTGCCATGTGGTTGAAT	CTTTGCATGATGGCGTCTCG
P-Selectin	GAAGTGTGACGCTGTGCAAT	CAGCTGGAGTCGTAGGCAAA
PECAM-1	GGAAGTGTCCTCCCTTGAGC	GGAGCCTTCCGTTCTTAGGG
eNOS	GCTCCCAACTGGACCATCTC	TCTTGCACGTAGGTCTTGGG
Ahr	TAAAGTCCACCCCTGCTGAC	CATTCAGCGCCTGTAACAAGA
Keap1	GGCAGGACCAGTTGAACAGT	CATAGCCTCCGAGGACGTAG
RelA	CCTGGAGCAAGCCATTAGC	CGCACTGCATTCAAGTCATAG
IKK-β	GTGCCTGTGACAGCTTACCT	CTCCAGTCTAGAGTCGTGAAGC
IL-6	CCCCAATTTCCAATGCTCTCC	CGCACTAGGTTTGCCGAGTA
TNF-α	ATGGCCTCCCTCTCATCAGT	TTTGCTACGACGTGGGCTAC
RPL32	TAAGCGAAACTGGCGGAAAC	CATCAGGATCTGGCCCTTGA
B2M	ACTGACCGGCCTGTATGCTA	CAATGTGAGGCGGGTGGAA

ICAM-1—Intercellular adhesion molecule 1; VCAM-1—Vascular cell adhesion molecule 1; E-Selectin—Selectin, endothelial cell; P-Selectin—Selectin platelet; PECAM-1—Platelet/endothelial cell adhesion molecule 1; eNOS—Nitric oxide synthase endothelial cell; Ahr—Aryl-hydrocarbon receptor; Keap1—Kelch-like ECH-associated protein 1; RelA—Transcription factor p65; IKK-β—Inhibitor of nuclear factor kappa-B kinase subunit beta; IL-6—Interleukin 6; TNF-α—Tumor necrosis factor α; RPL32—Ribosomal protein L32; B2M—Beta-2 microglobulin.

## Data Availability

All data are described in the manuscript.
